# Leukocyte Chemotactic Factor 2 Amyloidosis (LECT‐2) Amyloidosis Case Report and Review of the Current Literature

**DOI:** 10.1002/ccr3.72055

**Published:** 2026-02-12

**Authors:** Ahmad Samir Matarneh, Bayan Matarneh, Omar K. Salameh, Sundus Sardar, Catherine Abendroth, Nasrollah Ghahramani, Navin Verma

**Affiliations:** ^1^ Department of Nephrology Penn State Milton S Hershey Medical Center Hershey Pennsylvania USA; ^2^ DMC, Children's Hospital Detroit Michigan USA; ^3^ University Health Truman Medical Center Kansas City MIssouri USA; ^4^ Department of Pathology Penn State Milton S Hershey Medical Center Hershey Pennsylvania USA

**Keywords:** ALECT2 amyloidosis, amyloid typing, chronic kidney disease, nephrotic‐range proteinuria, renal biopsy

## Abstract

Amyloid deposition is an increasingly recognized contributor to chronic kidney disease and end‐stage renal disease. It can result from various underlying conditions, including monoclonal gammopathies and chronic inflammatory states. Diagnosis is typically established by kidney biopsy demonstrating characteristic amyloid deposits. ALECT2 (leukocyte chemotactic factor 2) amyloidosis can present as nephrotic‐range proteinuria. ALECT2 amyloidosis is an uncommon and under‐recognized.

## 1 Introduction

Renal amyloidosis is a well‐established cause of proteinuria and progressive kidney dysfunction, resulting from the deposition of misfolded proteins in the renal parenchyma [1]. Several types of amyloid proteins have been identified, with varying organ involvement and clinical implications. Among these, amyloid associated with leukocyte chemotactic factor 2 (ALECT2) represents a rare and often underrecognized subtype. ALECT2 amyloidosis primarily involves the kidneys, liver, and spleen, and although it is most frequently reported in individuals of Hispanic descent, it has been described in diverse populations worldwide [2].

Early and accurate diagnosis is essential, as it can prevent unnecessary treatments and guide supportive management strategies. Immunosuppressive therapies, which may be considered in other forms of renal amyloidosis, have not shown benefit in ALECT2. Currently, no standardized treatment guidelines exist, and management focuses on controlling associated complications. Diagnosis relies on histopathologic confirmation, typically through kidney biopsy [3]. Given its potential to involve multiple organs, ALECT2 amyloidosis can present with a wide spectrum of clinical manifestations, which makes it important to consider in patients with unexplained proteinuria and chronic kidney disease [4].

We report a case of ALECT2 amyloidosis identified on kidney biopsy in a patient presenting with nephrotic‐range proteinuria, an uncommon manifestation of this disease.

## 2 Case History and Examination

A 76‐year‐old Egyptian man with a medical history notable for hypertension, well‐controlled on amlodipine, was referred to the nephrology clinic for evaluation of newly identified nephrotic‐range proteinuria. A 24‐h urine collection quantified proteinuria at 3.68 g per day. He denied edema, hematuria, constitutional symptoms, or systemic signs suggestive of a secondary cause of nephrotic syndrome.

Initial laboratory studies showed a serum creatinine of 1.31 mg/dL and estimated glomerular filtration rate (eGFR) of 57, with no hematuria on urinalysis. Autoimmune workup, including antinuclear antibody (ANA), antineutrophil cytoplasmic antibodies (ANCA), complement levels (C3 and C4), and anti‐double‐stranded DNA antibodies, was unremarkable. Serum protein electrophoresis and immunofixation revealed no evidence of monoclonal gammopathy, and serum free light chains were within normal limits, reducing the likelihood of light chain (AL) amyloidosis. Of note, his liver function was normal.

## 3 Differential Diagnosis, Investigations, and Treatment

**FIGURE 1:**
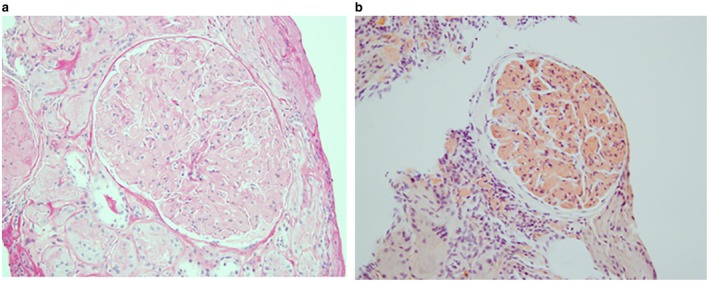
(a) Homogeneous PAS‐negative material expanding mesangial stalk (PAS, ×500); (b) Congo‐red positive staining of mesangial matrical material (Congo‐Red, ×500).

**FIGURE 2:**
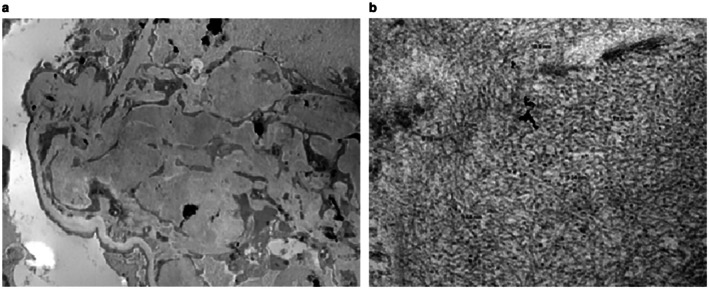
(a) Expansion of mesangial stalk by electron dense deposits (transmission electron microscopy, ×2800); (b) material comprised of randomly oriented fibrils of 11 nm average diameter (transmission electron microscopy, ×55,000).

In view of the unexplained nephrotic‐range proteinuria, a percutaneous kidney biopsy was performed. Light microscopy demonstrated marked expansion of glomerular mesangial stalks by homogeneous matrix that was pale on PAS and silver stains and demonstrated apple‐green birefringence under polarized light examination of Congo Red stain, consistent with amyloid deposition (Figure 1a,b). There was relatively minimal interstitial and peri‐arteriolar deposition of Congo‐Red positive material. Immunofluorescence demonstrated weak diffuse staining for IgG in the expanded mesangial stalks, with polyclonal kappa and lambda light chain staining. Electron microscopy revealed expansive mesangial deposition of 10 nm diameter fibrillar material in randomly oriented arrays (Figure 2a,b). Mass spectrometry analysis identified the deposits as leukocyte chemotactic factor 2 (ALECT2) amyloid.

## 4 Conclusions and Results

The patient was referred to hematology for further assessment, which excluded an associated plasma cell disorder. No involvement of other organs was identified on clinical evaluation. Following diagnosis, the patient was managed conservatively with optimization of blood pressure control and initiation of renin–angiotensin system blockade. Over approximately 6 months of follow‐up, kidney function improved, with serum creatinine decreasing to 1.0 mg/dL and estimated glomerular filtration rate increasing to 73 mL/min/1.73 m^2^, where it subsequently plateaued. Proteinuria demonstrated a modest reduction on repeat assessment. No evidence of extrarenal amyloid involvement was identified during follow‐up.

## 5 Discussion

ALECT2 amyloidosis is a rare and underdiagnosed form of amyloid disease first identified as a distinct entity in 2008 through advances in proteomic analysis. Unlike the more common forms, such as AL (light chain) and AA (serum amyloid A protein) amyloidosis, ALECT2 is not linked to plasma cell disorders, chronic inflammatory diseases, or known inherited mutations [5]. This distinction is clinically important because it has direct implications for diagnosis and management.

The exact cause of ALECT2 amyloidosis is not well understood. It is thought that abnormal folding of the LECT2 protein leads to the formation of amyloid fibrils, which accumulate in the extracellular matrix. The kidneys, particularly the cortical interstitium and vasculature, and the liver are the most commonly affected organs [6]. The reason why certain individuals develop LECT2 deposits while others do not is unclear. Genetic factors, environmental influences, or changes in protein processing may contribute, but further research is needed.

Patients with ALECT2 amyloidosis are typically older adults. The disease often presents with slowly progressive chronic kidney disease and low‐grade proteinuria. Nephrotic‐range proteinuria, although less frequent than in AL amyloidosis, can occur, as in our case. Hematuria is usually absent. Hematuria is usually absent, in addition to the kidneys and liver, ALECT2 amyloidosis frequently involves the spleen and adrenal glands among extrarenal sites [7]. These features can delay diagnosis because they differ from the more systemic presentation seen in AL amyloidosis.

ALECT2 amyloidosis shows a striking ethnic pattern. It has been reported most often in people of Hispanic background, especially in the southwestern United States, where it is now recognized as a common cause of renal amyloidosis. However, cases have also been described in people of Middle Eastern, Egyptian, Punjabi, and Native American ancestry, suggesting that the disease may be more widespread than originally thought [8].

Accurate diagnosis relies on kidney biopsy. On microscopy, amyloid deposits are usually found in the interstitium and small vessels, though glomerular involvement may be present. Congo red staining confirms the presence of amyloid through its characteristic apple‐green birefringence under polarized light [9]. However, identifying the amyloid type is essential because treatment strategies differ. Mass spectrometry is the most reliable method for typing, as immunohistochemistry can sometimes give inconclusive results [10].

Distinguishing ALECT2 from other forms of amyloidosis is crucial. AL amyloidosis, for example, would prompt a search for plasma cell dyscrasia and consideration of chemotherapy, which is ineffective and potentially harmful in ALECT2. Similarly, AA amyloidosis would lead to evaluation for an inflammatory source and use of anti‐inflammatory treatment, which is not beneficial in this context [8].

Management of ALECT2 amyloidosis is supportive. There is no specific treatment to halt or reverse the amyloid deposition. Blood pressure control, ideally with renin‐angiotensin system blockade, and reduction of proteinuria are the main goals. Although the progression is generally slow, kidney function can decline over time, and some patients will eventually require dialysis [9].

In contrast to AL and AA amyloidosis, kidney injury in ALECT2 appears to evolve through gradual structural involvement rather than an aggressive or inflammatory process. Amyloid deposition predominantly affects the interstitium, vasculature, and mesangium, leading to progressive nephron loss over time [11, 12]. This pattern is consistent with reports describing ALECT2 as a cause of slowly progressive chronic kidney disease, often with preserved renal function at presentation. While nephrotic‐range proteinuria is less frequently reported, its presence may reflect more extensive glomerular involvement and could be associated with a higher risk of progression, although data remain limited [13, 14].

This case highlights the importance of a thorough evaluation in older adults with nephrotic‐range proteinuria, especially when initial investigations rule out common causes. Early kidney biopsy and accurate amyloid typing help ensure that patients receive appropriate care and avoid unnecessary treatments. Greater recognition of ALECT2 amyloidosis will improve diagnosis and contribute to a better understanding of its distribution and natural history. Short‐term improvement in kidney function, as observed in this patient, has been described in ALECT2 amyloidosis and likely reflects hemodynamic optimization and reduction in intraglomerular pressure rather than regression of amyloid deposition.

Further studies are needed to clarify the mechanisms that drive LECT2 misfolding and amyloid formation. Identifying risk factors and understanding why certain populations are affected could eventually lead to the development of targeted therapies or preventive measures [15]. We summarized the existing literature with cases reporting ALECT‐2 amyloidosis in Table 1.

**TABLE 1:** Published reports of ALECT2 amyloidosis.

Author (Year)	Country/Population	No. of patients	Presentation	Extrarenal involvement	Key findings
Benson et al. (2008) [2]	USA	1 (initial case)	CKD with nephrotic range proteinuria	None explicitly described	First description of LECT2 as a novel renal amyloid protein.
Said et al. (2014) [8]	USA (Hispanic‐majority)	72	Slowly progressive CKD, modest proteinuria	Extrarenal involvement was present in some patients	Characterization and outcomes; ALECT2 identified as the third most common renal amyloid type.
Larsen et al. (2016) [15]	Egypt	(Case series)	CKD in general renal amyloidosis cohort	—	Showed ALECT2 accounted for ~31% of renal amyloid cases in Egyptians.
Xu et al. (2022) [12]	China	1	CKD + concurrent IgA nephropathy	None described	Case of renal amyloidogenic LECT2 plus IgA nephropathy.
Nasr et al. (2021) [13]	USA (transplant allograft)	1	Kidney transplant graft dysfunction	Donor kidney involvement	Case of donor‐derived ALECT2 amyloidosis in renal transplant.

This case highlights the importance of considering ALECT2 amyloidosis in the differential diagnosis of nephrotic‐range proteinuria, particularly when autoimmune and hematologic evaluations are unremarkable. Timely kidney biopsy with accurate amyloid typing is essential to establish the diagnosis, guide appropriate management, and avoid unnecessary or potentially harmful treatments.

## Author Contributions

**Ahmad Samir Matarneh:** conceptualization, writing – original draft, writing – review and editing. **Bayan Matarneh:** conceptualization, writing – original draft, writing – review and editing. **Omar K. Salameh:** writing – original draft. **Sundus Sardar:** writing – original draft, writing – review and editing. **Catherine Abendroth:** data curation, writing – original draft. **Nasrollah Ghahramani:** conceptualization, writing – original draft, writing – review and editing. **Navin Verma:** writing – original draft, writing – review and editing.

## Funding

The funding process was performed solely by the writing authors.

## Ethics Statement

Ethical approval for this study was waived by the Penn State Health Ethical Committee because this is a case report.

## Consent

Written informed consent was obtained from the patient for publication of this case report and any accompanying images. A copy of the written consent is available for review by the Editor‐in‐Chief of this journal.

## Conflicts of Interest

The authors associated with this case report have no actual or possible conflicts of interest to declare.

## Data Availability

The data supporting the findings of this study are available from the corresponding author upon reasonable request.
